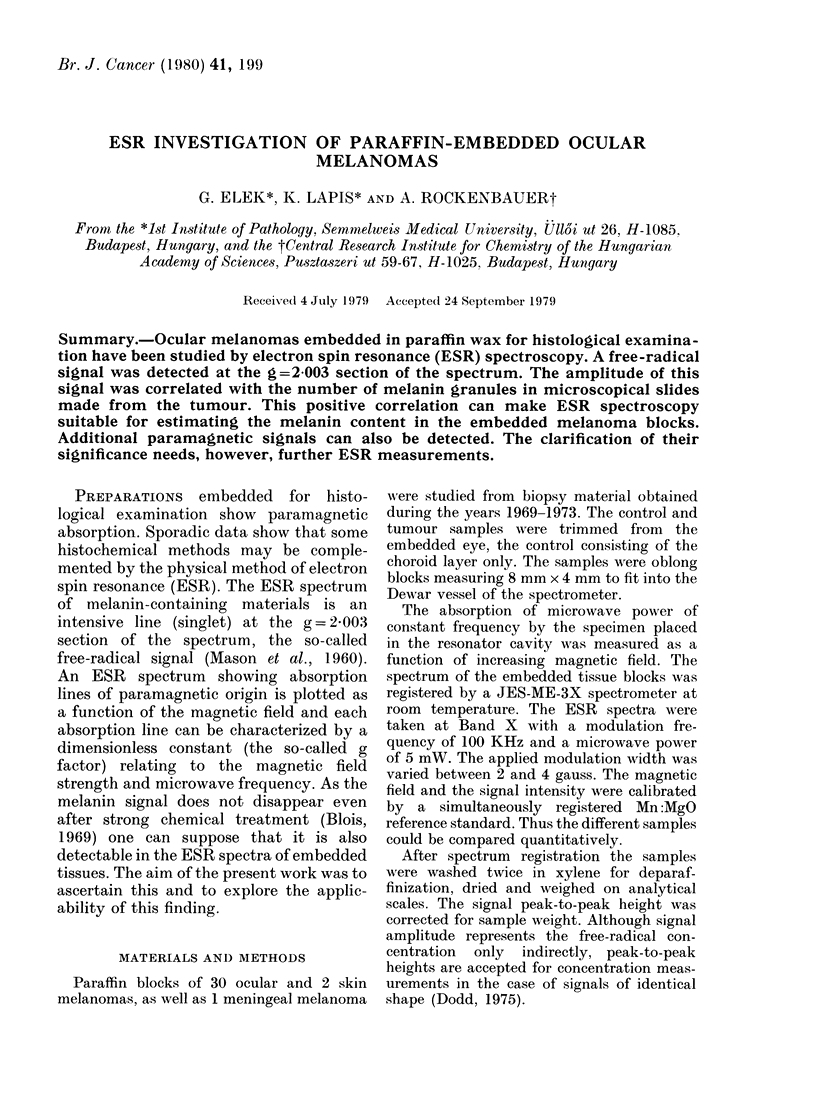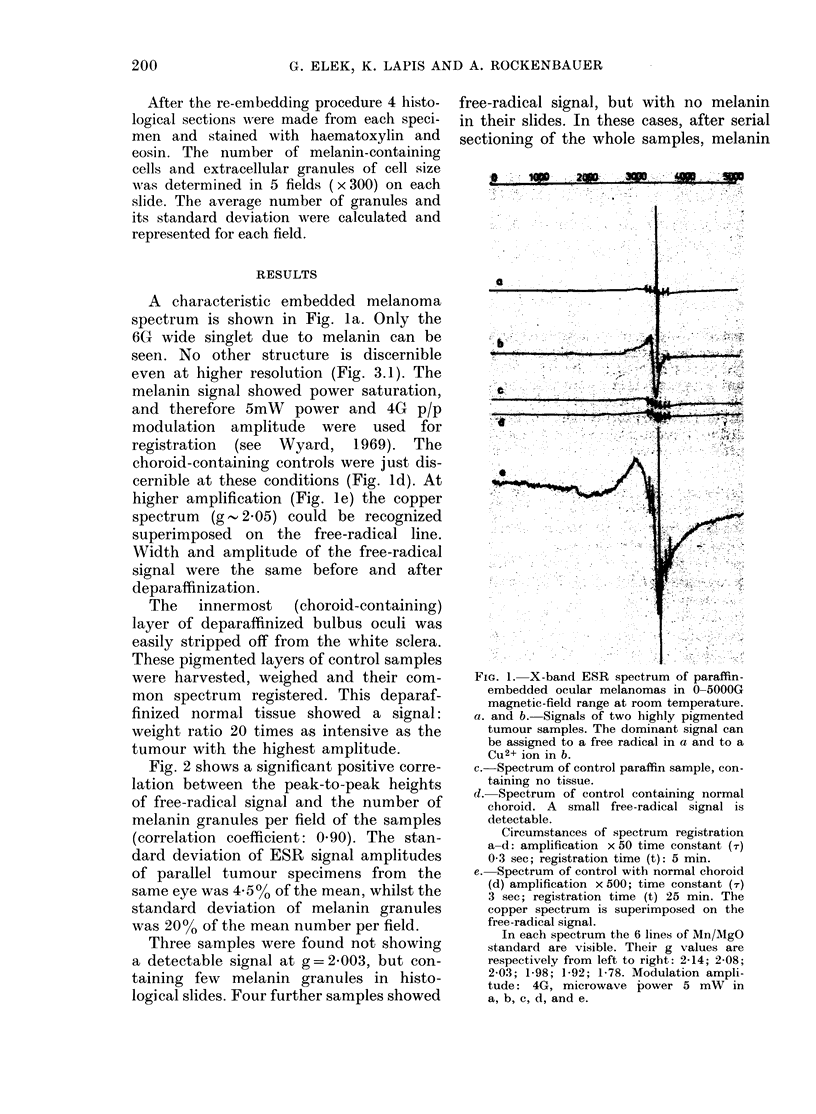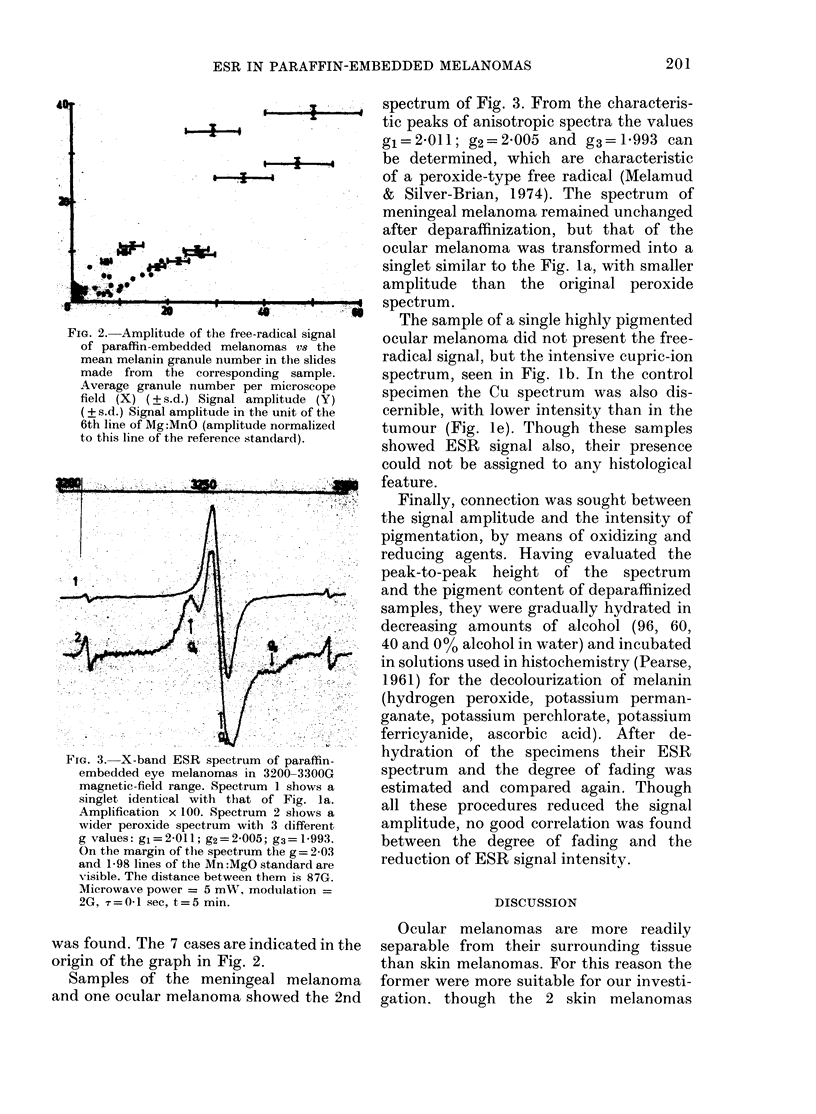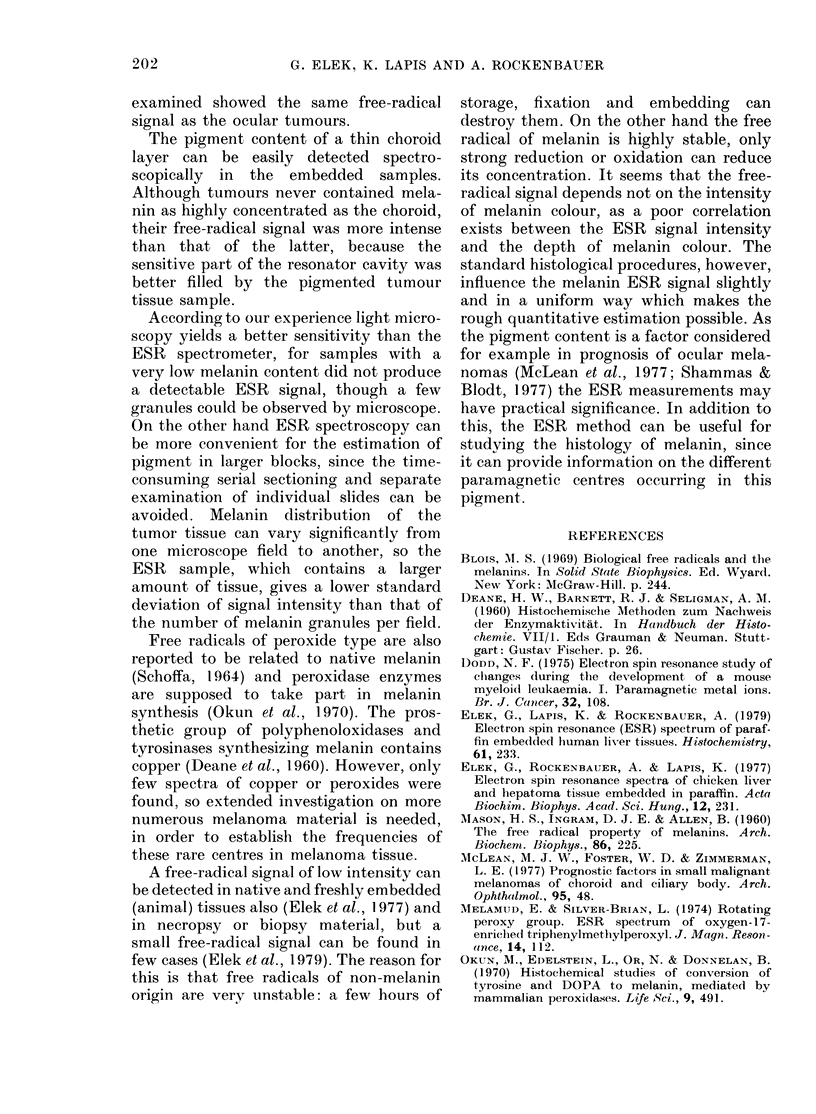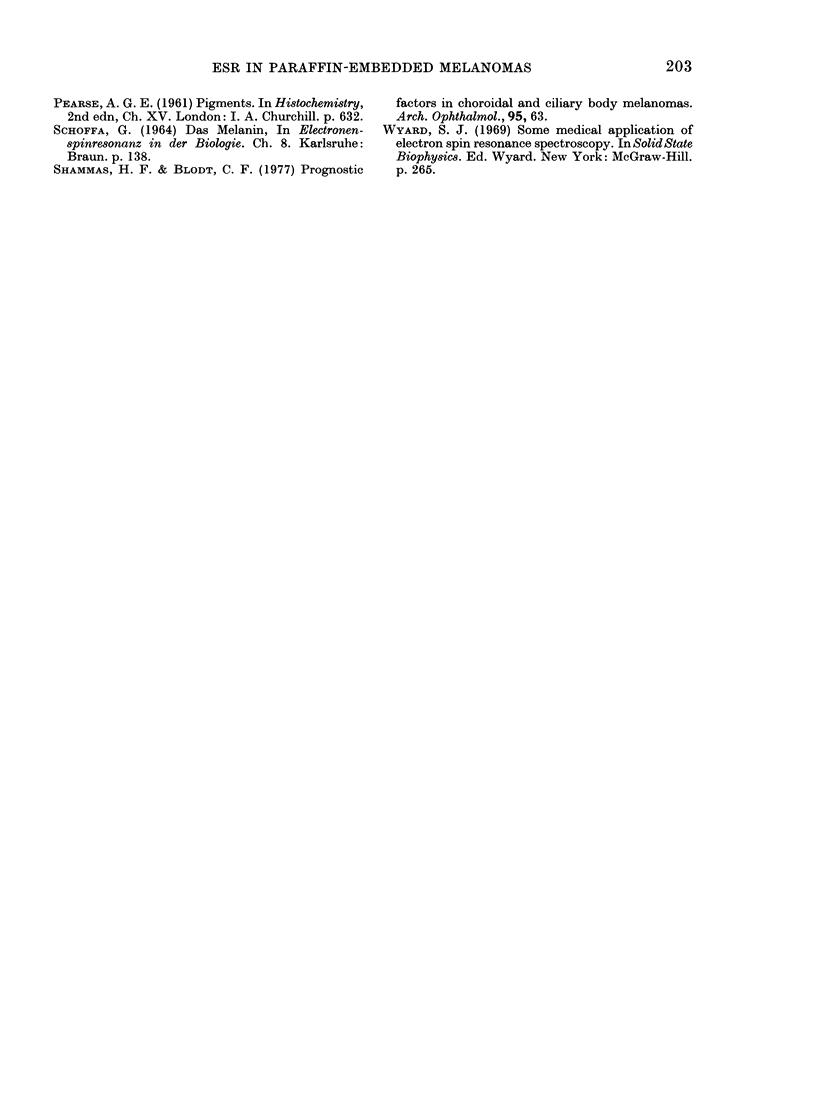# ESR investigation of paraffin-embedded ocular melanomas.

**DOI:** 10.1038/bjc.1980.30

**Published:** 1980-02

**Authors:** G. Elek, K. Lapis, A. Rockenbauer

## Abstract

Ocular melanomas embedded in paraffin wax for histological examination have been studied by electron spin resonance (ESR) spectroscopy. A free-radical signal was detected at the g = 2.003 section of the spectrum. The amplitude of this signal was correlated with the number of melanin granules in microscopical slides made from the tumour. This positive correlation can make ESR spectroscopy suitable for estimating the melanin content in the embedded melanoma blocks. Additional paramagnetic signals can also be detected. The clarification of their significance needs, however, further ESR measurements.


*Br. J. Cancer* (1980) **41**, 199

### ESR INVESTIGATION OF PARAFFIN-EMBEDDED OCULAR MELANOMAS

G. ELEK*, K. LAPIS* AND A. ROCKENBAUER†

*From the *1st Institute of Pathology, Semmelweis Medical University, Üllői ut* 26, *H*-1085, *Budapest, Hungary, and the †Central Research Institute for Chemistry of the Hungarian Academy of Sciences, Pusztaszeri ut* 59-67, *H*-1025, *Budapest, Hungary*

Received 4 July 1979 Accepted 24 September 1979

**Summary.—Ocular melanomas embedded in paraffin wax for histological examination have been studied by electron spin resonance (ESR) spectroscopy. A free-radical signal was detected at the g=2·003 section of the spectrum. The amplitude of this signal was correlated with the number of melanin granules in microscopical slides made from the tumour. This positive correlation can make ESR spectroscopy suitable for estimating the melanin content in the embedded melanoma blocks. Additional paramagnetic signals can also be detected. The clarification of their significance needs, however, further ESR measurements.**

PREPARATIONS embedded for histological examination show paramagnetic absorption. Sporadic data show that some histochemical methods may be complemented by the physical method of electron spin resonance (ESR). The ESR spectrum of melanin-containing materials is an intensive line (singlet) at the $g = 2{\cdot}003$ section of the spectrum, the so-called free-radical signal (Mason *et al.*, 1960). An ESR spectrum showing absorption lines of paramagnetic origin is plotted as a function of the magnetic field and each absorption line can be characterized by a dimensionless constant (the so-called g factor) relating to the magnetic field strength and microwave frequency. As the melanin signal does not disappear even after strong chemical treatment (Blois, 1969) one can suppose that it is also detectable in the ESR spectra of embedded tissues. The aim of the present work was to ascertain this and to explore the applicability of this finding.

### MATERIALS AND METHODS

Paraffin blocks of 30 ocular and 2 skin melanomas, as well as 1 meningeal melanoma were studied from biopsy material obtained during the years 1969–1973. The control and tumour samples were trimmed from the embedded eye, the control consisting of the choroid layer only. The samples were oblong blocks measuring 8 mm × 4 mm to fit into the Dewar vessel of the spectrometer.

The absorption of microwave power of constant frequency by the specimen placed in the resonator cavity was measured as a function of increasing magnetic field. The spectrum of the embedded tissue blocks was registered by a JES-ME-3X spectrometer at room temperature. The ESR spectra were taken at Band X with a modulation frequency of 100 KHz and a microwave power of 5 mW. The applied modulation width was varied between 2 and 4 gauss. The magnetic field and the signal intensity were calibrated by a simultaneously registered Mn:MgO reference standard. Thus the different samples could be compared quantitatively.

After spectrum registration the samples were washed twice in xylene for deparaffinization, dried and weighed on analytical scales. The signal peak-to-peak height was corrected for sample weight. Although signal amplitude represents the free-radical concentration only indirectly, peak-to-peak heights are accepted for concentration measurements in the case of signals of identical shape (Dodd, 1975).

After the re-embedding procedure 4 histological sections were made from each specimen and stained with haematoxylin and eosin. The number of melanin-containing cells and extracellular granules of cell size was determined in 5 fields ($\times 300$) on each slide. The average number of granules and its standard deviation were calculated and represented for each field.

RESULTS

A characteristic embedded melanoma spectrum is shown in Fig. 1a. Only the 6G wide singlet due to melanin can be seen. No other structure is discernible even at higher resolution (Fig. 3.1). The melanin signal showed power saturation, and therefore 5mW power and 4G p/p modulation amplitude were used for registration (see Wyard, 1969). The choroid-containing controls were just discernible at these conditions (Fig. 1d). At higher amplification (Fig. 1e) the copper spectrum ($g \sim 2{\cdot}05$) could be recognized superimposed on the free-radical line. Width and amplitude of the free-radical signal were the same before and after deparaffinization.

The innermost (choroid-containing) layer of deparaffinized bulbus oculi was easily stripped off from the white sclera. These pigmented layers of control samples were harvested, weighed and their common spectrum registered. This deparaffinized normal tissue showed a signal: weight ratio 20 times as intensive as the tumour with the highest amplitude.

Fig. 2 shows a significant positive correlation between the peak-to-peak heights of free-radical signal and the number of melanin granules per field of the samples (correlation coefficient: 0·90). The standard deviation of ESR signal amplitudes of parallel tumour specimens from the same eye was 4·5% of the mean, whilst the standard deviation of melanin granules was 20% of the mean number per field.

Three samples were found not showing a detectable signal at $g = 2{\cdot}003$, but containing few melanin granules in histological slides. Four further samples showed free-radical signal, but with no melanin in their slides. In these cases, after serial sectioning of the whole samples, melanin

FIG. 1.—X-band ESR spectrum of paraffin-embedded ocular melanomas in 0–5000G magnetic-field range at room temperature.

*a.* and *b.*—Signals of two highly pigmented tumour samples. The dominant signal can be assigned to a free radical in *a* and to a $Cu^{2+}$ ion in *b*.

*c.*—Spectrum of control paraffin sample, containing no tissue.

*d.*—Spectrum of control containing normal choroid. A small free-radical signal is detectable.

Circumstances of spectrum registration a–d: amplification $\times 50$ time constant ($\tau$) 0·3 sec; registration time (t): 5 min.

*e.*—Spectrum of control with normal choroid (d) amplification $\times 500$; time constant ($\tau$) 3 sec; registration time (t) 25 min. The copper spectrum is superimposed on the free-radical signal.

In each spectrum the 6 lines of Mn/MgO standard are visible. Their g values are respectively from left to right: 2·14; 2·08; 2·03; 1·98; 1·92; 1·78. Modulation amplitude: 4G, microwave power 5 mW in a, b, c, d, and e.

FIG. 2.—Amplitude of the free-radical signal of paraffin-embedded melanomas *vs* the mean melanin granule number in the slides made from the corresponding sample. Average granule number per microscope field (X) ($\pm$s.d.) Signal amplitude (Y) ($\pm$s.d.) Signal amplitude in the unit of the 6th line of Mg:MnO (amplitude normalized to this line of the reference standard).

FIG. 3.—X-band ESR spectrum of paraffin-embedded eye melanomas in 3200–3300G magnetic-field range. Spectrum 1 shows a singlet identical with that of Fig. 1a. Amplification $\times$100. Spectrum 2 shows a wider peroxide spectrum with 3 different g values: $g_1 = 2{\cdot}011$; $g_2 = 2{\cdot}005$; $g_3 = 1{\cdot}993$. On the margin of the spectrum the $g = 2{\cdot}03$ and 1·98 lines of the Mn:MgO standard are visible. The distance between them is 87G. Microwave power = 5 mW, modulation = 2G, $\tau = 0{\cdot}1$ sec, t = 5 min.

was found. The 7 cases are indicated in the origin of the graph in Fig. 2.

Samples of the meningeal melanoma and one ocular melanoma showed the 2nd spectrum of Fig. 3. From the characteristic peaks of anisotropic spectra the values $g_1 = 2{\cdot}011$; $g_2 = 2{\cdot}005$ and $g_3 = 1{\cdot}993$ can be determined, which are characteristic of a peroxide-type free radical (Melamud & Silver-Brian, 1974). The spectrum of meningeal melanoma remained unchanged after deparaffinization, but that of the ocular melanoma was transformed into a singlet similar to the Fig. 1a, with smaller amplitude than the original peroxide spectrum.

The sample of a single highly pigmented ocular melanoma did not present the free-radical signal, but the intensive cupric-ion spectrum, seen in Fig. 1b. In the control specimen the Cu spectrum was also discernible, with lower intensity than in the tumour (Fig. 1e). Though these samples showed ESR signal also, their presence could not be assigned to any histological feature.

Finally, connection was sought between the signal amplitude and the intensity of pigmentation, by means of oxidizing and reducing agents. Having evaluated the peak-to-peak height of the spectrum and the pigment content of deparaffinized samples, they were gradually hydrated in decreasing amounts of alcohol (96, 60, 40 and 0% alcohol in water) and incubated in solutions used in histochemistry (Pearse, 1961) for the decolourization of melanin (hydrogen peroxide, potassium permanganate, potassium perchlorate, potassium ferricyanide, ascorbic acid). After dehydration of the specimens their ESR spectrum and the degree of fading was estimated and compared again. Though all these procedures reduced the signal amplitude, no good correlation was found between the degree of fading and the reduction of ESR signal intensity.

### DISCUSSION

Ocular melanomas are more readily separable from their surrounding tissue than skin melanomas. For this reason the former were more suitable for our investigation. though the 2 skin melanomas examined showed the same free-radical signal as the ocular tumours.

The pigment content of a thin choroid layer can be easily detected spectroscopically in the embedded samples. Although tumours never contained melanin as highly concentrated as the choroid, their free-radical signal was more intense than that of the latter, because the sensitive part of the resonator cavity was better filled by the pigmented tumour tissue sample.

According to our experience light microscopy yields a better sensitivity than the ESR spectrometer, for samples with a very low melanin content did not produce a detectable ESR signal, though a few granules could be observed by microscope. On the other hand ESR spectroscopy can be more convenient for the estimation of pigment in larger blocks, since the time-consuming serial sectioning and separate examination of individual slides can be avoided. Melanin distribution of the tumor tissue can vary significantly from one microscope field to another, so the ESR sample, which contains a larger amount of tissue, gives a lower standard deviation of signal intensity than that of the number of melanin granules per field.

Free radicals of peroxide type are also reported to be related to native melanin (Schoffa, 1964) and peroxidase enzymes are supposed to take part in melanin synthesis (Okun *et al.*, 1970). The prosthetic group of polyphenoloxidases and tyrosinases synthesizing melanin contains copper (Deane *et al.*, 1960). However, only few spectra of copper or peroxides were found, so extended investigation on more numerous melanoma material is needed, in order to establish the frequencies of these rare centres in melanoma tissue.

A free-radical signal of low intensity can be detected in native and freshly embedded (animal) tissues also (Elek *et al.*, 1977) and in necropsy or biopsy material, but a small free-radical signal can be found in few cases (Elek *et al.*, 1979). The reason for this is that free radicals of non-melanin origin are very unstable: a few hours of storage, fixation and embedding can destroy them. On the other hand the free radical of melanin is highly stable, only strong reduction or oxidation can reduce its concentration. It seems that the free-radical signal depends not on the intensity of melanin colour, as a poor correlation exists between the ESR signal intensity and the depth of melanin colour. The standard histological procedures, however, influence the melanin ESR signal slightly and in a uniform way which makes the rough quantitative estimation possible. As the pigment content is a factor considered for example in prognosis of ocular melanomas (McLean *et al.*, 1977; Shammas & Blodt, 1977) the ESR measurements may have practical significance. In addition to this, the ESR method can be useful for studying the histology of melanin, since it can provide information on the different paramagnetic centres occurring in this pigment.

### REFERENCES

BLOIS, M. S. (1969) Biological free radicals and the melanins. In *Solid State Biophysics*. Ed. Wyard. New York: McGraw-Hill. p. 244.

DEANE, H. W., BARNETT, R. J. & SELIGMAN, A. M. (1960) Histochemische Methoden zum Nachweis der Enzymaktivität. In *Handbuch der Histochemie*. VII/1. Eds Grauman & Neuman. Stuttgart: Gustav Fischer. p. 26.

DODD, N. F. (1975) Electron spin resonance study of changes during the development of a mouse myeloid leukaemia. I. Paramagnetic metal ions. *Br. J. Cancer*, **32**, 108.

ELEK, G., LAPIS, K. & ROCKENBAUER, A. (1979) Electron spin resonance (ESR) spectrum of paraffin embedded human liver tissues. *Histochemistry*, **61**, 233.

ELEK, G., ROCKENBAUER, A. & LAPIS, K. (1977) Electron spin resonance spectra of chicken liver and hepatoma tissue embedded in paraffin. *Acta Biochim. Biophys. Acad. Sci. Hung.*, **12**, 231.

MASON, H. S., INGRAM, D. J. E. & ALLEN, B. (1960) The free radical property of melanins. *Arch. Biochem. Biophys.*, **86**, 225.

MCLEAN, M. J. W., FOSTER, W. D. & ZIMMERMAN, L. E. (1977) Prognostic factors in small malignant melanomas of choroid and ciliary body. *Arch. Ophthalmol.*, **95**, 48.

MELAMUD, E. & SILVER-BRIAN, L. (1974) Rotating peroxy group. ESR spectrum of oxygen-17-enriched triphenylmethylperoxyl. *J. Magn. Resonance*, **14**, 112.

OKUN, M., EDELSTEIN, L., OR, N. & DONNELAN, B. (1970) Histochemical studies of conversion of tyrosine and DOPA to melanin, mediated by mammalian peroxidases. *Life Sci.*, **9**, 491.

PEARSE, A. G. E. (1961) Pigments. In *Histochemistry*, 2nd edn, Ch. XV. London: I. A. Churchill. p. 632.
SCHOFFA, G. (1964) Das Melanin, In *Electronen-spinresonanz in der Biologie*. Ch. 8. Karlsruhe: Braun. p. 138.
SHAMMAS, H. F. & BLODT, C. F. (1977) Prognostic factors in choroidal and ciliary body melanomas. *Arch. Ophthalmol.*, **95**, 63.
WYARD, S. J. (1969) Some medical application of electron spin resonance spectroscopy. In *Solid State Biophysics*. Ed. Wyard. New York: McGraw-Hill. p. 265.